# Seroprevalence of *Toxoplasma gondii* in slaughtered horses and donkeys in Liaoning province, northeastern China

**DOI:** 10.1186/1756-3305-6-140

**Published:** 2013-05-16

**Authors:** Na Yang, Ming-Yang Mu, Gao-ming Yuan, Guo-xin Zhang, Hong-Kui Li, Jian-Bin He

**Affiliations:** 1 College of Animal Science and Veterinary Medicine, Shenyang Agricultural University, Shenyang, Liaoning Province 110866, China; 2 Liaoning Center for Animal Disease Control and Prevention, Shenyang, Liaoning Province 110164, China

**Keywords:** *Toxoplasma gondii*, Seroprevalence, Horse, Donkey, MAT

## Abstract

**Background:**

*Toxoplasma gondii* is an important zoonotic pathogen infecting humans and almost all warm-blooded animals. The most common sources of human infection are ingestion of tissue cysts in raw or undercooked meat. However, limited information is available about *T. gondii* infection in horses and donkeys in China. In the present study, we report the seroprevalence of *T. gondii* infection in horses and donkeys in Liaoning province, northeastern China.

**Methods:**

Serum samples were collected from 711 and 738 slaughtered horses and donkeys, respectively, in 13 regions of Liaoning province. The modified agglutination test (MAT) was used to test the specific antibodies to *T. gondii*.

**Results:**

In this study, 178 of 711 (25.0%) horses were seropositive for *T. gondii* with titers of 1:25 in 81, 1:50 in 62, 1:100 in 33, and 1:200 in 2, and seroprevalence of *T. gondii* infection from 13 regions ranged from 8.2% to 37.0%. Antibodies to *T. gondii* were found in 174 of 738 (23.6%) donkeys with titers of 1:25 in 66, 1:50 in 54, 1:100 in 49, and 1:200 in 5, and seroprevalence varied in 13 different regions, ranging from 9.1% to 32.6%.

**Conclusions:**

The results of the present study indicated that the rate of infection with *T. gondii* in horses and donkeys is a little high in Liaoning province, northeastern China in comparison to other surveys in China, which suggests that consumption of horse and donkey meat in this area may represent a potential source for human infection with *T. gondii*.

## Background

*Toxoplasma gondii* parasites are obligate intracellular apicomplexans that can infect virtually all warm-blooded animals; felids are definitive hosts. The most common sources of human infection are ingestion of tissue cysts in raw or undercooked meat or of food or water contaminated with oocysts shed by felids and transplacental transmission [1]. *T. gondii* infection may cause serious disease in pregnant women and immunocompromised patients, such as AIDS patients and organ transplant recipients [2-6].

Meat from birds and warm-blooded animals traditionally has been considered a major source of *T. gondii* infection [7]. Viable parasites have been isolated from the meat of these animals, such as sheep, goat, cow, pig, chicken and horse [8]. Horse meat and donkey meat are also important foodstuff worldwide, and humans can acquire infections with *T. gondii* by ingesting raw or undercooked horse and donkey meat containing tissue cysts.

In equids, *T. gondii* infections generally display a subclinical course, and serological techniques for the detection of specific antibodies produced in the body against the parasite have a great diagnostic value [8]. Serum antibodies to *T. gondii* have been found in horses and donkeys in many surveys worldwide [8]. Table 1 summarizes the reports of *T. gondii* infection in horses and donkeys from China because these papers were published in the Chinese language in local journals and are not easily accessible to foreign scholars. In view of this background, the objective of this study was to survey the seroprevalence of *T. gondii* infection in horses and donkeys in Liaoning province, northeastern China.

**Table 1:** **Seroprevalence of *T. gondii *infection in horses and donkeys from China**

**Province**	**Animal**	**No. tested**	**Positive(%)**	**Method**	**Year of sampling**	**Reference**
Xinjiang	Horse	60	0 (0)	IHA	<2002	[9]
	Donkey	30	0 (0)	IHA		
Hebei	Horse	65	4 (6.2)	ELISA	2000-2001	[10]
	Donkey	133	8 (8.6)	ELISA		
Shandong	Horse	147	0 (0)	IHA	<2001	[11]
	Donkey	17	1 (5.9)	IHA		
Qinghai	Horse	149	4 (2.7)	IHA	<2008	[12]
	Horse	314	19 (6.1)	IHA	2010	[13]

## Methods

### The study area

Liaoning province is located in northeastern China, its geographical position is at east longitude 118°50′ - 125°47′ and at north latitude 38°43′ - 43°29′. It has an area of 145,900 km^2^ and a population of approximately 44 million. The area has a temperate monsoon climate with abundant sunshine, a long winter, hot summer, a short spring and autumn. The annual average temperature is 7–11°C, with a highest temperature of 40°C and a lowest temperature of −30°C.

### Blood samples

Blood samples were collected from 711 and 738 slaughtered horses and donkeys, respectively, in 13 regions of Liaoning province, including Shenyang, Dalian, Anshan, Fushun, Benxi, Dandong, Jinzhou, Yingkou, Fuxin, Liaoyang, Panjin, Chaoyang, and Huludao between May and October 2012. Horses were >10-yr-old, 39 (21.9%) were female and 139 (78.1%) were male; and donkeys were approximately 1-yr-old, 21 (12.1%) were female and 153 (87.9%) were male. These equids fed on forage, and most of these equids had outdoor access. The information was obtained via personal interviews with the veterinarians and owners. Serum samples were separated and stored at −20°C until use.

### Serological examination

Sera were tested for *T. gondii* antibodies using 2-fold serial dilutions from 1:25 to 1:3,200 with the modified agglutination test (MAT), as described previously [14]. Briefly, the harvested parasites were kept in 6% formaldehyde solution at 4°C overnight, and suspended in the alkaline buffer at 20,000 parasites/mL. Two-fold dilutions of sera were performed using the serum diluting buffer, and agglutination was performed in U-bottom 96-well microtiter plates using a mixture of 50 μL antigen and 50 μL diluted sera. The plates were incubated at 37°C overnight. The test was considered positive when a layer of agglutinated parasites was formed in wells at dilutions of 1:25 or higher; positive and negative controls were included in each test.

### Statistical analysis

Statistical analysis of *T. gondii* seroprevalence between horse and donkey was performed using a Chi square test with SPSS (SPSS Inc., Chicago, Illinois). A *P*-value < 0.05 was considered statistically significant.

### Ethics statement

All animals were handled in strict accordance with good animal practice according to the Animal Ethics Procedures and Guidelines of the People's Republic of China, and the study was approved by the Animal Ethics Committee of Shenyang Agricultural University (Permit No. SYXK < Liao > 2011-0001).

## Results and discussion

In the present study, 178 of 711 (25.0%) horses were seropositive for *T. gondii*, with titers of 1:25 in 81, 1:50 in 62, 1:100 in 33, and 1:200 in 2 (Table 2), and seroprevalence of *T. gondii* infection from 13 regions ranged from 8.2% to 37.0% (Table 3). Antibodies to *T. gondii* were found in 174 of 738 (23.6%) donkeys, with titers of 1:25 in 66, 1:50 in 54, 1:100 in 49, and 1:200 in 5 (Table 2). Among these positive donkeys, seroprevalence varied in 13 different regions, ranging from 9.1% to 32.6% (Table 3). There was no significant difference between horse and donkey in Liaoning province (P>0.05).

**Table 2:** **Antibody titers of *T. gondii *infection in horses and donkeys in Liaoning province, northeastern China by modified agglutination test (MAT)**

	**No. of sera with MAT in different titers**				
**Animal**	**1:25**	**1:50**	**1:100**	**1:200**	**≥1:25**
Horse	81	62	33	2	178
Donkey	66	54	49	5	174
Total	147	116	82	7	352

**Table 3:** **The seroprevalence of *T. gondii *infection in horses and donkeys in Liaoning province, northeastern China**

**Region**	**Horse**		**Donkey**	
	**No. of sample**	**No. of positive (%)**	**No. of sample**	**No. of positive (%)**
Shenyang	48	15(31.3)	56	17(30.4)
Dalian	52	13(25.0)	48	11(22.9)
Anshan	70	18(25.7)	50	13(26.0)
Fushun	61	17(27.9)	64	19(29.7)
Benxi	54	20(37.0)	46	15(32.6)
Dandong	68	11(16.2)	71	12(16.9)
Jinzhou	55	7(12.7)	68	10(14.7)
Yingkou	40	13(32.5)	52	15(28.8)
Fuxin	78	22(28.2)	76	19(25.0)
Liaoyang	58	19(32.7)	48	15(31.3)
Panjin	45	15(33.3)	62	17(27.4)
Chaoyang	33	4(12.1)	42	6(14.3)
Huludao	49	4(8.2)	55	5(9.1)
Total	711	178(25.0)	738	174(23.6)

Some serological tests, including the Sabin-Feldman dye test (DT), latex agglutination test (LAT), indirect hemagglutination (IHA), indirect fluorescent antibody (IFA), enzyme-linked immunoabsorbent assay (ELISA), and modified agglutination test (MAT), have been used to detect *T. gondii* antibodies in equids; the latter (MAT) is both sensitive and specific [8]. Worldwide seroprevalence of *T. gondii* infection in horses has been summarised prior to 2010 by Dubey, ranging from 0.4% to 48.1% [8]; and since then, it has been reported to be 24% in the Czech Republic by LAT [15], 32% in Riyadh Province, Saudi Arabia by DT [16], 34.0% in Costa Rica by MAT [17], 69.6% in the Fernando de Noronha, Brazil by MAT [18], and 10.8% in Southern Spain by MAT [19]. Worldwide seroprevalence of *T. gondii* infection in donkeys was reported as 65.6% in Egypt by ELISA [8], 11.0% in Gemlik, Turkey by LAT [8], 25.6% in Southern Spain by MAT [19], and 43.2% in Northeast of Brazil by indirect immunofluorescence reaction (IFI**)**[20].

In China, some surveys have previously reported prevalence of *T. gondii* infection in horses and donkeys (Table 1), however, it is difficult to compare results of the present study with other surveys in China because of different serological tests used and due to the different regions of China studied. Most surveys used the IHA test, and one survey used ELISA. In this study, we used the MAT for the detection of *T. gondii* seroprevalence in horses and donkeys because it is the major recommended test for diagnosis of *T. gondii* infection in several animals and man [8,14]. MAT has high sensitivity and specificity among all serologic methods, and this method is cheaper, easier than other tests and does not need special sophisticated equipment [21,22]. In addition, many surveys in China were conducted many years ago.

In this study, we found that equids with outdoor access may have a greater chance of being exposed to the food and water contaminated with *T. gondii* oocysts, which had been excreted in feces by cats and might be present in forage and water. This is the reason that seroprevalence of *T. gondii* infection in equids was relatively high in the present study.

Results of the present study indicated that *T. gondii* infection is common in horses and donkeys in Liaoning province, and the parasite will remain present in their tissues for life. Horse meat and donkey meat were mostly consumed by local people. Therefore, animals such as horse and donkey are at a high risk of infection and act as a transmission route to humans. Further research on the role of equid meat in human infections and the pathogenesis of *T. gondii* is needed.

In this study, the seroprevalence results of *T. gondii* infection in equids were higher than other food animals, including pig, cattle, sheep, chicken, duck, and goose in Liaoning province, northeasern China (Table 4) [22-24]. These results also confirm that *T. gondii* infection in food animals is widespread in northeastern China and should be of public health concern.

**Table 4:** **The seroprevalence of *T. gondii *infection in other food animals, including pig, cattle, sheep, chicken, duck, and goose in Liaoning province, northeastern China**

**Animal**	**No. of sample**	**No. of positive (%)**	**Method**	**Reference**
Pig	1164	140 (12.0)	IHA	[23]
cattle	646	39 (6.0)	IHA	[23]
sheep	566	25 (4.4)	IHA	[24]
chicken	502	37 (5.8)	MAT	[22]
duck	268	26 (7.8)	MAT	[22]
goose	128	9 (4.7)	MAT	[22]

## Conclusions

The results of the present study indicated that the rate of infection with *T. gondii* in horses and donkeys is a little high in Liaoning province, northeastern China, which suggests that consumption of horse and donkey meat in this area may represent a potential source for human infection with *T. gondii*. Therefore, it is necessary to take integrated strategies, including efficient management measures to prevent and control *T. gondii* infection in horses and donkeys, which would help to reduce *T. gondii* infection in humans.

## Competing interests

The authors declare that they have no competing interests.

## Authors’ contributions

JBH and NY conceived and designed the study, and critically revised the manuscript. NY, MYM, GMY, GXZ and HKL performed the experiments, analysed the data and drafted the manuscript. All authors read and approved the final manuscript.
